# With No Lysine Kinase 1 Promotes Metabolic Derangements and RV Dysfunction in Pulmonary Arterial Hypertension

**DOI:** 10.1016/j.jacbts.2021.09.004

**Published:** 2021-11-22

**Authors:** Sasha Z. Prisco, Megan Eklund, Rashmi Raveendran, Thenappan Thenappan, Kurt W. Prins

**Affiliations:** Lillehei Heart Institute, Cardiovascular Division, Department of Medicine, University of Minnesota Medical School, Minneapolis, Minnesota, USA

**Keywords:** lipotoxicity, metabolism, mitochondria, pulmonary arterial hypertension, right ventricular dysfunction, with no lysine kinase 1, AMPK, adenosine monophosphate-activated protein kinase, AS160, 160 kDa substrate of the Akt serine/threonine kinase, DCA, dicarboxylic fatty acid, FAO, fatty acid oxidation, GLO1, glyoxalase 1, GLO2, glyoxalase 2, GLUT1, glucose transporter 1, GLUT4, glucose transporter 4, LV, left ventricle/ventricular, MCT, monocrotaline, MCT-V, monocrotaline-vehicle, PAH, pulmonary arterial hypertension, PTM, post-translationally modify/modifications, PV, pressure-volume, PVR, pulmonary vascular resistance, RA, right atrial, RV, right ventricle/ventricular, RVD, right ventricular dysfunction, Tau/τ, right ventricular relaxation time, TCA, tricarboxylic acid, UDP-GlcNAC, uridine diphosphate *N*-acetylglucosamine, WNK, with no lysine kinase

## Abstract

Small molecule inhibition of with no lysine kinase 1 (WNK1) (WNK463) signaling activates adenosine monophosphate-activated protein kinase signaling and mitigates membrane enrichment of glucose transporters 1 and 4, which decreases protein O-GlcNAcylation and glycation. Quantitative proteomics of right ventricular (RV) mitochondrial enrichments shows WNK463 prevents down-regulation of several mitochondrial metabolic enzymes. and metabolomics analysis suggests multiple metabolic processes are corrected. Physiologically, WNK463 augments RV systolic and diastolic function independent of pulmonary arterial hypertension severity. Hypochloremia, a condition of predicted WNK1 activation in patients with pulmonary arterial hypertension, is associated with more severe RV dysfunction. These results suggest WNK1 may be a druggable target to combat metabolic dysregulation and may improve RV function and survival in pulmonary arterial hypertension.

## Highlights

• Small molecule inhibition of WNK1 (WNK463) signaling mitigates upregulation of the membrane glucose channels GLUT1 and GLUT4, restores levels of several glucose metabolites, and decreases protein O-GlcNAcylation and glycation in the RV.
• Quantitative proteomics of RV mitochondrial enrichments shows WNK463 treatment prevents down-regulation of several mitochondrial enzymes in the tricarboxylic acid cycle, fatty acid oxidation pathway, and the electron transport chain complexes.
• Integration of proteomics and metabolomics analysis suggests WNK463 reduces glutaminolysis induction and lipotoxicity caused by impaired mitochondrial fatty acid oxidation.
• WNK463 augments RV systolic and diastolic function independent of PAH severity.
• Hypochloremia, a condition of predicted WNK1 activation in patients with PAH, is associated with more severe RV dysfunction.

## Visual Abstract

**Figure:**
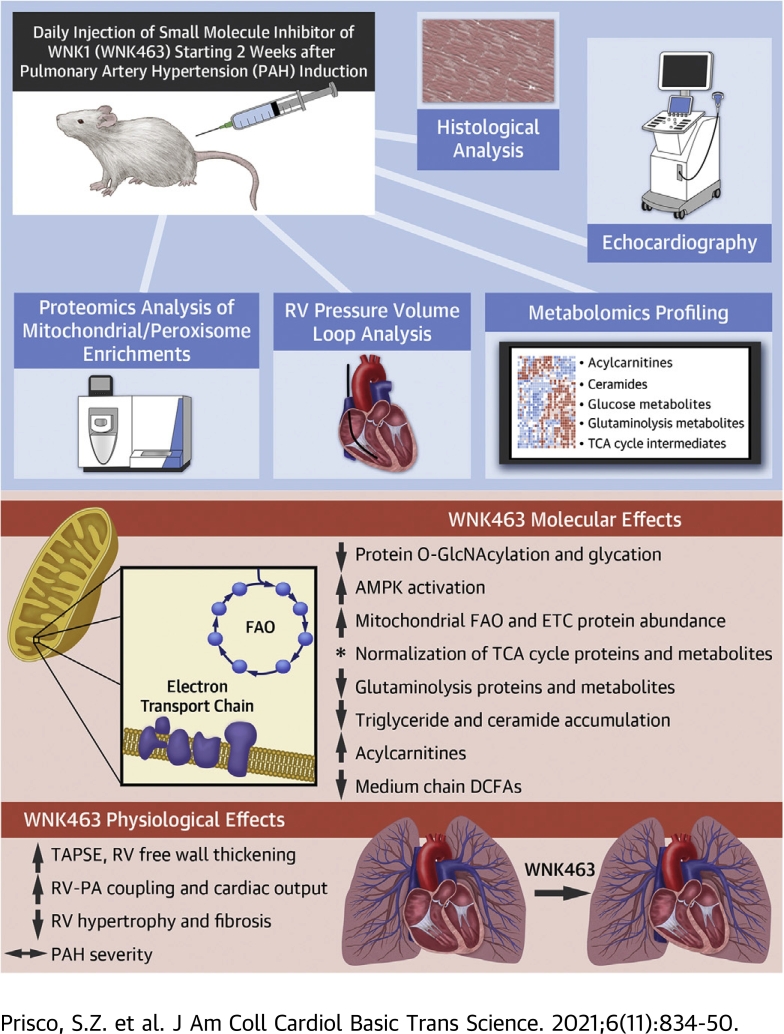

Pulmonary arterial hypertension (PAH) is a proliferative vasculopathy of the resistance pulmonary arteries that leads to elevated pulmonary arterial pressures (1). The pathological changes in the pulmonary vascular bed augment the workload of the right ventricle (RV), which ultimately results in RV dysfunction (RVD) (2,3). Metabolic derangements are the most robustly characterized phenotypes of RVD in PAH (4,5), with resultant increases in RV glucose uptake in both preclinical (6,7) and human PAH (8,9). RV function is inversely associated with RV glucose uptake (8), which implies excess intracellular glucose may have deleterious effects. Although glucose metabolism primarily generates adenosine triphosphate, the hexosamine biosynthetic pathway converts glucose to UDP-*N*-acetylglucosamine (UDP-GlcNAc) (10). UDP-GlcNAc is used to enzymatically post-translationally modify (PTM) serine or tyrosine residues, a process known as protein O-GlcNAcylation (10). Moreover, 1%-2% of glucose metabolized through glycolysis results in formation of methylglyoxal, a highly reactive dicarbonyl that can nonenzymatically modify proteins (protein glycation) (11). Both excess O-GlcNAcylation and glycation promote left ventricular (LV) cardiomyocyte mitochondrial dysfunction (12,13), but the role of these PTMs in RVD is relatively unexplored.

Two clinical studies showed hypochloremia identified high-risk patients with PAH, which might have direct relevance to glucose metabolism. Naal et al (14) showed that hypochloremia was independently associated with increased mortality. We also demonstrated that hypochloremia was independently associated with increased mortality and measures of RV failure in a multicenter study (15). The with-no-lysine (WNK) kinase proteins are a family of signaling kinases activated in the setting of low intracellular chloride levels (16,17). In the heart, WNK1 is the predominant isoform expressed (17), but studies of WNK1 in cardiac diseases are lacking. WNK1 function is better understood in skeletal muscle because previous studies showed WNK1 promoted membrane localization of the glucose channels, glucose transporter (GLUT)1 (18) and GLUT4 (19) via activation of 160-kDa substrate of the Akt Ser/Thr kinase (AS160), a Ras-related in brain GTPase-activating protein. In addition to modulation of glucose handling, inhibition of WNK1 increases phosphorylation and subsequent activation of adenosine monophosphate-activated protein kinase (AMPK) (20), a regulator of metabolism via mitochondrial and peroxisomal biogenesis (21,22). Thus, these data suggest hypochloremia could result in WNK1 activation and subsequently modulate RV metabolism via glucose handling and AMPK activation.

We investigated the effects of WNK inhibition on RV glucose handling and glucose-mediated PTMs, mitochondrial/peroxisomal density, and protein regulation, global metabolism, and function in preclinical PAH. We implemented a translational approach by treating monocrotaline (MCT) rats 2 weeks after the development of PAH and comprehensively examined the response of the RV to WNK inhibition using immunoblots, quantitative confocal microscopy, proteomics, metabolomics, echocardiography, and pressure-volume (PV) loop analysis. Finally, because hypochloremia activates WNK1 (16,23,24), we assessed the association between hypochloremia and RV function in 217 patients with PAH.

## Methods

An expanded Methods section appears in the Supplemental Appendix. In brief, male Sprague Dawley rats (200-250 g; 7-8 weeks old) (Charles River Laboratories) received a single subcutaneous injection of MCT (60 mg/kg) or phosphate-buffered saline (control rats). Two weeks after MCT injection, rats were given daily intraperitoneal injections of either 3 mg/kg WNK463 or vehicle (5% propylene glycol, 0.475% Pluronic, 0.475% Klucel LF, and 94.05% water) (25) for 10 days. Immunoblots of RV extracts were completed as previously described (26). Antibodies used in this study are listed in Supplemental Table 1. Complete Western blot images are shown in Supplemental Figure 1.

RV mitochondrial/peroxisomal enrichment fractions were used for quantitative mass spectrometry as described in the Supplemental Appendix. Metabolomic profiling of frozen RV tissues was completed by Metabolon, Inc.

Lung and cardiac histology, echocardiography (Supplemental Figure 2), and PV loops (Supplemental Figure 3) quantified the effects of WNK463 on RV structure and/or function and pulmonary vascular disease severity.

Finally, we determined how hypochloremia affected RV function in a PAH cohort of 217 patients (Supplemental Table 2).

## Results

### WNK inhibition mitigated GLUT1 and GLUT4 upregulation and reduced levels of multiple RV glucose metabolites

**Figure 1:**
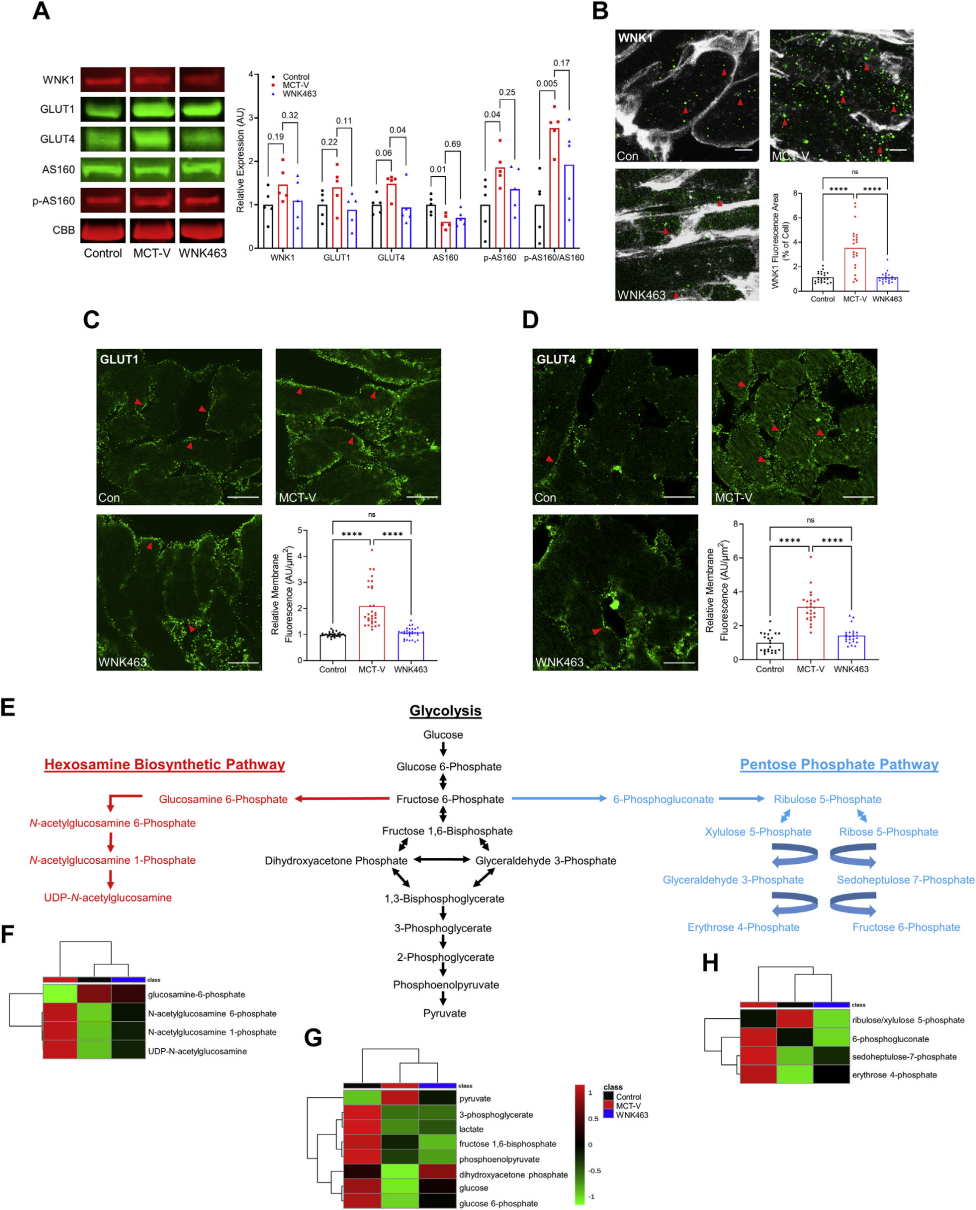
WNK Inhibition Combats RV Glucotoxicity in MCT Rats **(A)** Representative Western blots and quantification of protein abundance in right ventricular (RV) extracts from control, monocrotaline-vehicle (MCT-V), and WNK463 rats demonstrate WNK463 trends toward normalizing expression of WNK1 (control rats: 1.0 ± 0.4; MCT-V: 1.5 ± 0.4; WNK463: 1.1 ± 0.5 expression relative to control rats; n = 5 RVs per group for all proteins evaluated), glucose transporter 1 (GLUT1) (control rats: 1.0 ± 0.3; MCT-V: 1.4 ± 0.5; WNK463: 0.9 ± 0.4), GLUT4 (control rats: 1.0 ± 0.2; MCT-V: 1.5 ± 0.3; WNK463: 0.9 ± 0.4), 160 kDa substrate of the Akt serine/threonine kinase (AS160) (control rats: 1.0 ± 0.2; MCT-V: 0.6 ± 0.2; WNK463: 0.7 ± 0.2), phosphorylated AS160 (control rats: 1.0 ± 0.6; MCT-V: 1.9 ± 0.4; WNK463: 1.4 ± 0.5), and phosphorylated AS160 normalized to AS160 (control rats: 1.0 ± 0.7; MCT-V: 2.8 ± 0.5; WNK463: 1.9 ± 1.0). Western blot results were normalized to the myosin heavy chain band in the Coomassie brilliant blue (CBB) stained post-transfer gel. Values are expression relative to control rats. Representative immunofluorescence images from RV free wall sections show WNK463 significantly reduced the amount of **(B)** cytoplasmic WNK1 expression **(red arrows)** (wheat germ agglutinin staining in **white**, WNK1 staining in **green**) and **(C)** GLUT1 receptors **(red arrows)** and **(D)** GLUT4 receptors **(red arrows)** at the cell membrane. Scale bar 10 μm in **B** and 20 μm in **C and D**. n = 3 RVs per group and 7-10 areas assessed per RV in **B to D**. **(E)** Hexosamine biosynthetic pathway, glycolysis, and pentose phosphate pathway intermediates are outlined. RV metabolomics studies demonstrate WNK463 partially restores the levels of **(F)** hexosamine biosynthesis pathway, **(G)** glycolysis, and **(H)** pentose phosphate pathway intermediates as depicted by hierarchical cluster analysis. One-way analysis of variance (ANOVA) with Dunnett post hoc analysis was completed in **(A)**. ∗∗∗∗*P <* 0.0001, and NS by Brown-Forsythe and Welch ANOVA with Dunnett multiple comparison test in **B and C** and 1-way ANOVA with Tukey post hoc test in **D**.

Immunoblots showed MCT-Vehicle (MCT-V) RVs had elevated levels of WNK1, GLUT1, GLUT4, phosphorylated (active form) of AS160 (p-AS160), and the ratio of p-AS160/AS160, which was mitigated by WNK463 (Figure 1A). Confocal microscopy revealed significantly increased WNK1 immunoreactivity (Figure 1B) and membrane localization of GLUT1 (Figure 1C) and GLUT4 (Figure 1D) in MCT-V RVs. WNK463 significantly reduced WNK1 staining intensity (Figure 1B) and GLUT1 (Figure 1C) and GLUT4 membrane enrichment (Figure 1D). Metabolomics profiling quantified the effects of WNK inhibition on glucose metabolites in the hexosamine biosynthetic, glycolytic, and pentose phosphate pathways (Figure 1E). In MCT-V RVs, the levels of the end products in the hexosamine biosynthetic (UDP-GlcNAc) (Figure 1F), glycolytic (pyruvate) (Figure 1G), and pentose phosphate pathways (erythrose 4-phosphate) (Figure 1H) were all higher than that in control subjects, but WNK463 restored the concentration of these metabolites (Figures 1F and 1G). Thus, these data supported a role of WNK1 in regulating RV glucose handling and metabolism.

### WNK inhibition combated excess protein O-GlcNAcylation and glycation

**Figure 2:**
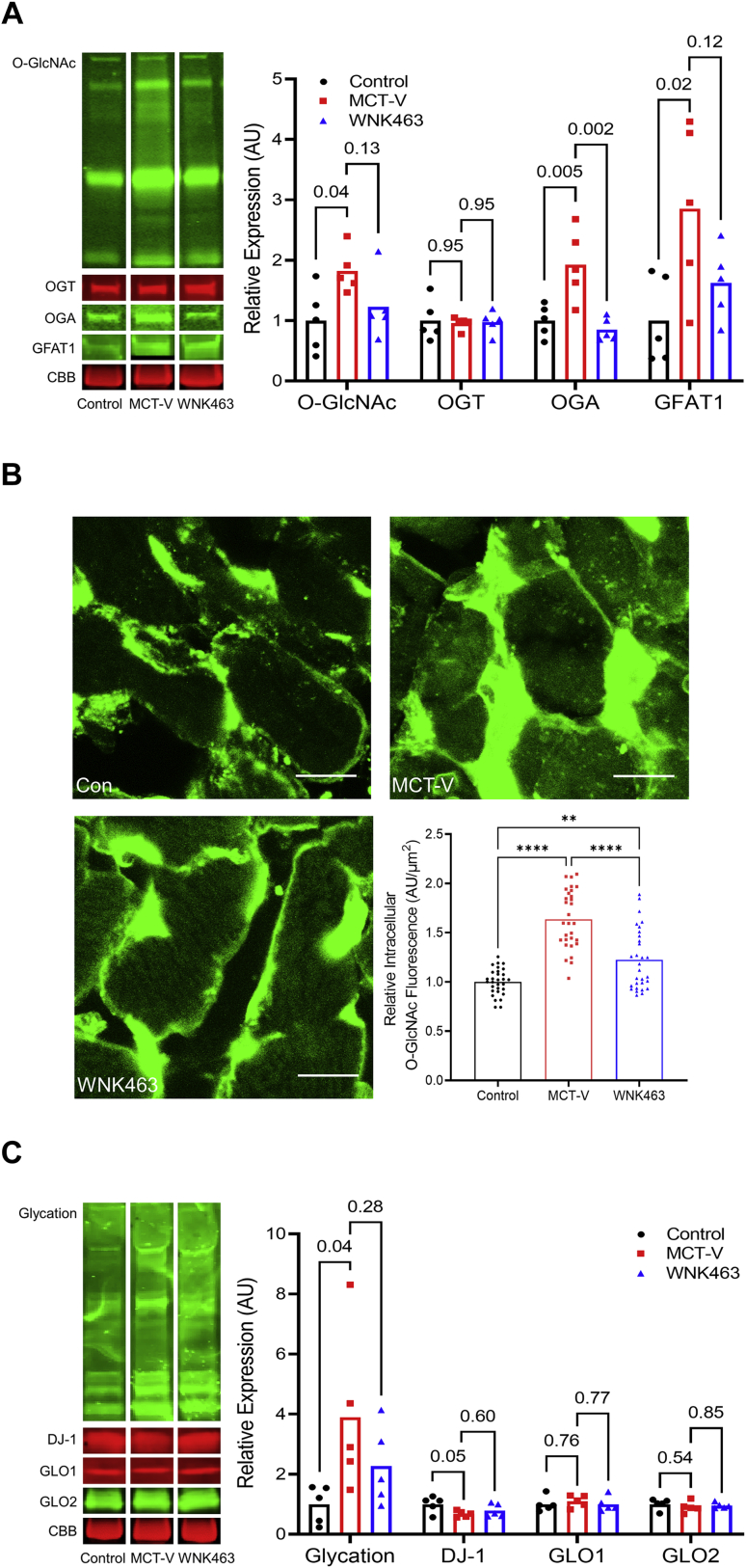
Protein O-Glcnacylation and Glycation Are Blunted by WNK463 **(A)** Representative Western blots and quantification of protein abundance in RV extracts from control, MCT-V, and WNK463 rats demonstrate WNK463 trends toward normalizing protein O-GlcNAcylation (control rats: 1.0 ± 0.5; MCT-V: 1.8 ± 0.4; WNK463: 1.2 ± 0.5 expression relative to control rats; n = 5 RVs per group for all proteins assessed), O-GlcNAcase (OGA) (control rats: 1.0 ± 0.3; MCT-V: 1.9 ± 0.6; WNK463: 0.9 ± 0.2), and glutamine-fructose-6-phosphate transaminase 1 (GFAT) (control rats: 1.0 ± 0.7; MCT-V: 2.9 ± 1.4; WNK463: 1.6 ± 0.6) expression and did not change O-linked β-*N*-acetylglucosamine transferase (OGT) abundance (control rats: 1.0 ± 0.3; MCT-V: 1.0 ± 0.1; WNK463: 1.0 ± 0.2). Western blot results were normalized to the myosin heavy chain band in the CBB stained post-transfer gel. Values are expression relative to control rats. **(B)** Representative confocal micrographs of RV free wall sections stained with succinylated wheat germ agglutinin (WGA) show increased intracellular O-GlcNAcylation signal in MCT-V, which is reduced by WNK463. Scale bar 10 μm. n = 3 RVs per group, 10 areas assessed per RV. **(C)** Representative Western blots and quantification of protein glycation, DJ-1, glyoxalase 1 (GLO1), and GLO2 reveal WNK463 nonsignificantly reduces total protein glycation (control rats: 1.0 ± 0.6; MCT-V: 3.9 ± 2.7; WNK463: 2.3 ± 1.3) without altering DJ-1, GLO1, and GLO2 abundance in the RV. One-way ANOVA with Dunnett post hoc analysis was completed in **A and C**. ∗∗*P <* 0.01 and ∗∗∗∗*P <* 0.0001 by Brown-Forsythe and Welch ANOVA with Dunnett multiple comparison test in **B**. Abbreviations as in Figure 1.

Next, we examined how WNK463 treatment altered protein O-GlcNAcylation/glycation in the RV. O-GlcNAcylation was increased in MCT-V RVs, which was mitigated by WNK463 treatment (Figure 2A). Furthermore, WNK463 reduced expression of O-GlcNAcase and glutamine-fructose-6-phosphate transaminase 1 (Figure 2A). Confocal microscopy showed WNK463 significantly decreased intracellular cardiomyocyte O-GlcNAcylation (Figure 2B). Next, we investigated the effects of WNK inhibition on protein glycation and the proteins that modulate protein glycation. WNK463 curtailed excess protein glycation that was observed in MCT-V without drastically changing expression of DJ-1, a protein deglycase, glyoxalase 1 (GLO1) and GLO2, proteins that catabolize methylglyoxal, the glucose metabolite responsible for protein glycation (Figure 2C) (27). Thus, WNK463 decreased protein O-GlcNAcylation/glycation.

### WNK463 Activated AMPK, Normalized Mitochondrial Density, and Partially Prevented Dysregulation of Mitochondrial Metabolic Proteins

**Figure 3:**
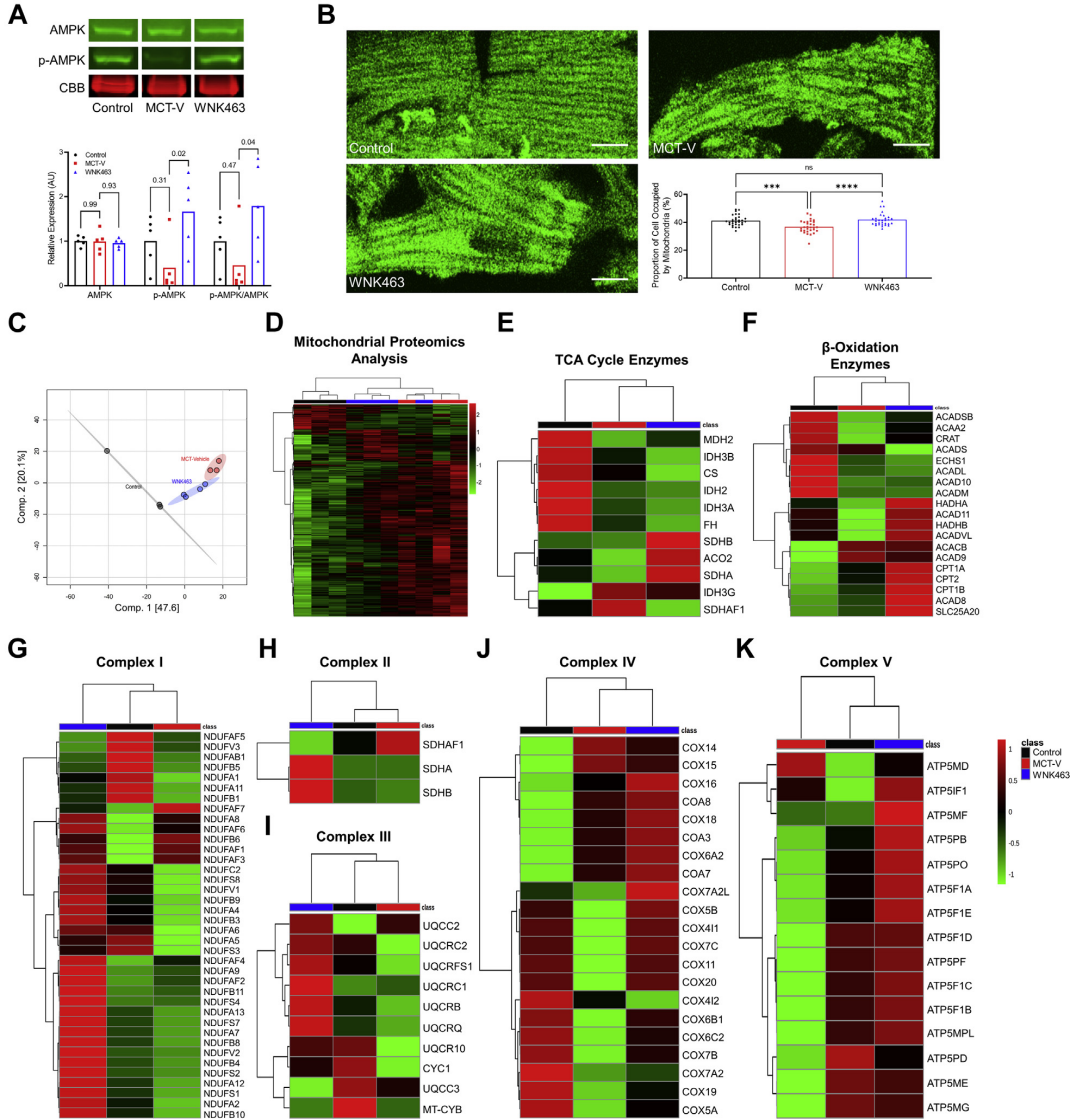
WNK463 Activates AMPK, Restores Mitochondrial Density, and Prevents Downregulation of Numerous Mitochondrial Metabolic Enzymes (**A**) Representative Western blots and quantification of protein abundance in RV extracts from control rats, MCT-V, and WNK463 rats reveal that WNK463 increases phosphorylated AMPK (control rats: 1.0 ± 0.6; MCT-V: 0.4 ± 0.6; WNK463: 1.7 ± 0.8 expression relative to control rats; n = 5 RVs per group for all proteins assessed) and the ratio of p-AMPK to AMPK abundance (control rats: 1.0 ± 0.6; MCT-V: 0.5 ± 0.7; WNK463: 1.8 ± 1.0) compared with MCT-V without changing AMPK expression. Western blot results were normalized to the myosin heavy chain band in the CBB stained post-transfer gel. Values are expression relative to control rats. **(B)** Representative confocal micrographs of RV cardiomyocytes stained with mitofusin-2 (MFN2) to assess mitochondrial density. WNK463 normalizes mitochondrial density. Scale bar 10 μm. n = 3 RVs per group;10 cardiomyocytes assessed per RV. **(C)** Principal component analysis and **(D)** hierarchical cluster analysis show WNK463 partially normalizes the global expression signature of mitochondrial/peroxisomal proteins. Hierarchical cluster analysis of **(E)** tricarboxylic acid (TCA) cycle enzymes, **(F)** fatty acid β-oxidation enzymes, **(G)** complex I, **(H)** complex II, **(I)** complex III, **(J)** complex IV, and **(K)** complex V proteins demonstrate WNK463 increases multiple mitochondrial metabolic proteins. One-way ANOVA with Dunnett post hoc analysis was completed in **A**. ∗∗∗*P <* 0.001; ∗∗∗∗*P <* 0.0001, and NS by 1-way ANOVA with Tukey post hoc analysis in **B**. Abbreviations as in Figure 1.

To further define how WNK1 inhibition modulated mitochondrial homeostasis, we evaluated AMPK activation, mitochondrial density, and defined mitochondrial protein regulation using quantitative proteomics. There was no difference in total AMPK expression in the RV when we compared control rats to MCT-V rats, but MCT-V rats had decreased p-AMPK levels and a lower ratio of p-AMPK/total AMPK. WNK463 treatment increased p-AMPK, and the p-AMPK/AMPK ratio was higher than levels in control rats (Figure 3A). Then, we evaluated mitochondrial density in RV cardiomyocytes using confocal microscopy. Compared to control rats, MCT-V had decreased mitochondrial density, which WNK463 corrected (Figure 3B). We next performed quantitative proteomic profiling to examine how WNK463 altered mitochondrial protein homeostasis. We identified 2,970 total proteins in our extracts, and 1,203 proteins had significant differences in abundance. Principal component analysis revealed WNK463 shifted the proteomic signature toward control rats (Figure 3C). Hierarchical cluster analysis corroborated this finding (Figure 3D). Next, we determined how WNK463 altered expression of tricarboxylic acid (TCA) cycle enzymes. WNK463 increased expression of the enzymes succinate dehydrogenase complex iron sulfur subunit B, aconitase 2, and succinate dehydrogenase complex flavoprotein subunit A compared with that of MCT-V (Figure 3E). Then, we probed mitochondrial fatty acid oxidation (FAO) enzyme regulation. MCT-V rats had lower levels of multiple FAO proteins, which WNK463 combated (Figure 3F). Finally, the protein subunits of the electron transport chain complexes I-V were evaluated. MCT-V RVs had a reduction in nearly all subunits of complexes I-V, but WNK463 elevated expression of most of these proteins (Figures 3G to 3K).

### Metabolomics profiling demonstrated WNK463 improved RV metabolism

**Figure 4:**
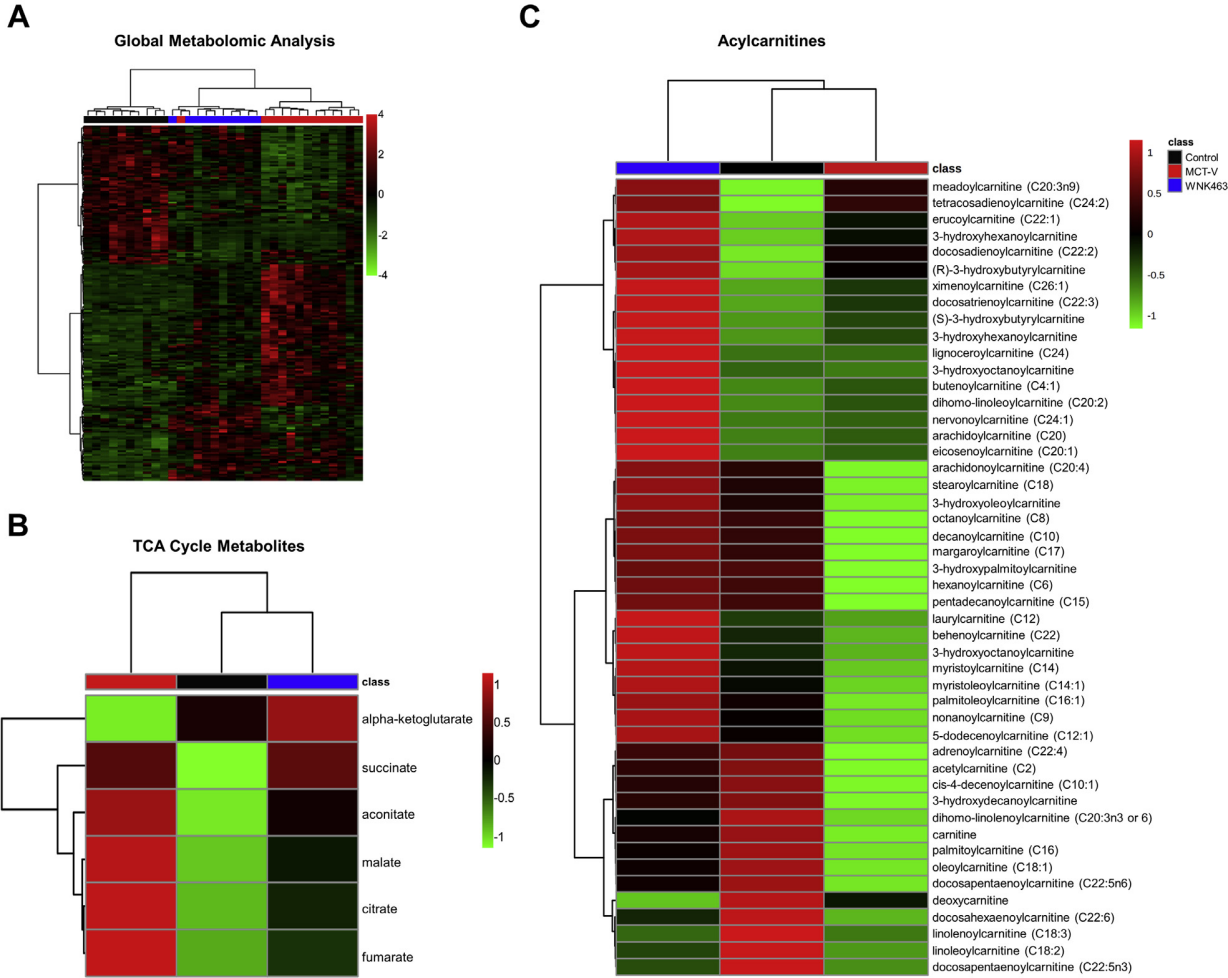
Global Metabolomics Profiling Shows WNK463 Partially Corrects RV Metabolic Derangements Hierarchical cluster analysis of **(A)** global RV metabolomics data, **(B)** TCA cycle metabolites, and **(C)** acylcarnitines. Abbreviations as in Figures 1 and 3.

Global metabolomic profiling examined the metabolic state of the RV in control, MCT-V, and WNK463 rats. WNK463 modulated the RV metabolic signature, with a pattern that was an intermediate between control and MCT-V rats in hierarchical cluster analysis (Figure 4A). As discussed previously, WNK463 prevented accumulation of pyruvate, suggesting less use of glycolysis (Figure 1G). We subsequently determined how WNK463 altered TCA cycle metabolites. Compared with RVs in control rats, MCT-V RVs had elevated levels of nearly all TCA metabolites, which WNK463 combated (Figure 4B). Next, we analyzed 48 acylcarnitine-associated metabolites to probe mitochondrial FAO. Consistent with a previous study (28), nearly all acylcarnitines were reduced in MCT-V RVs. However, WNK463 increased concentrations of most acylcarnitines even beyond that of RVs in control rats (Figure 4C). In conclusion, these data indicated WNK463 altered RV metabolism. In particular, the restoration of metabolites in glycolysis, the TCA cycle, and acylcarnitines suggested multiple metabolic pathways were enhanced by WNK antagonism.

### WNK463 mitigated secondary metabolic effects of impaired mitochondrial FAO

**Figure 5:**
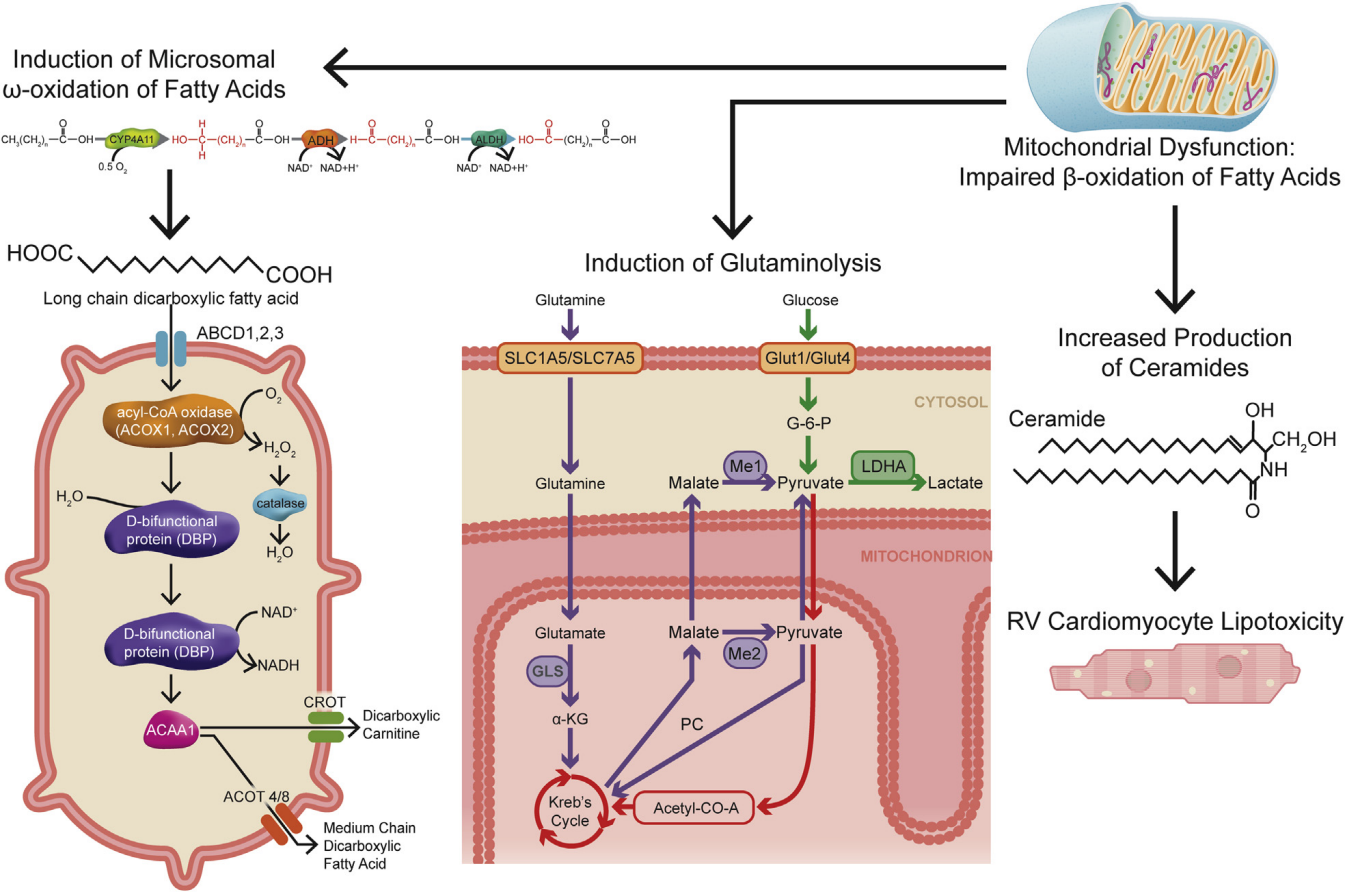
Diagram of the Metabolic Consequences of Impaired Mitochondrial β-FAO Three secondary metabolic consequences of altered mitochondrial fatty acid oxidation (FAO) include induction of ω-FAO, glutaminolysis, and lipotoxicity caused by ceramide accumulation. Abbreviations as in Figure 1.

**Figure 6:**
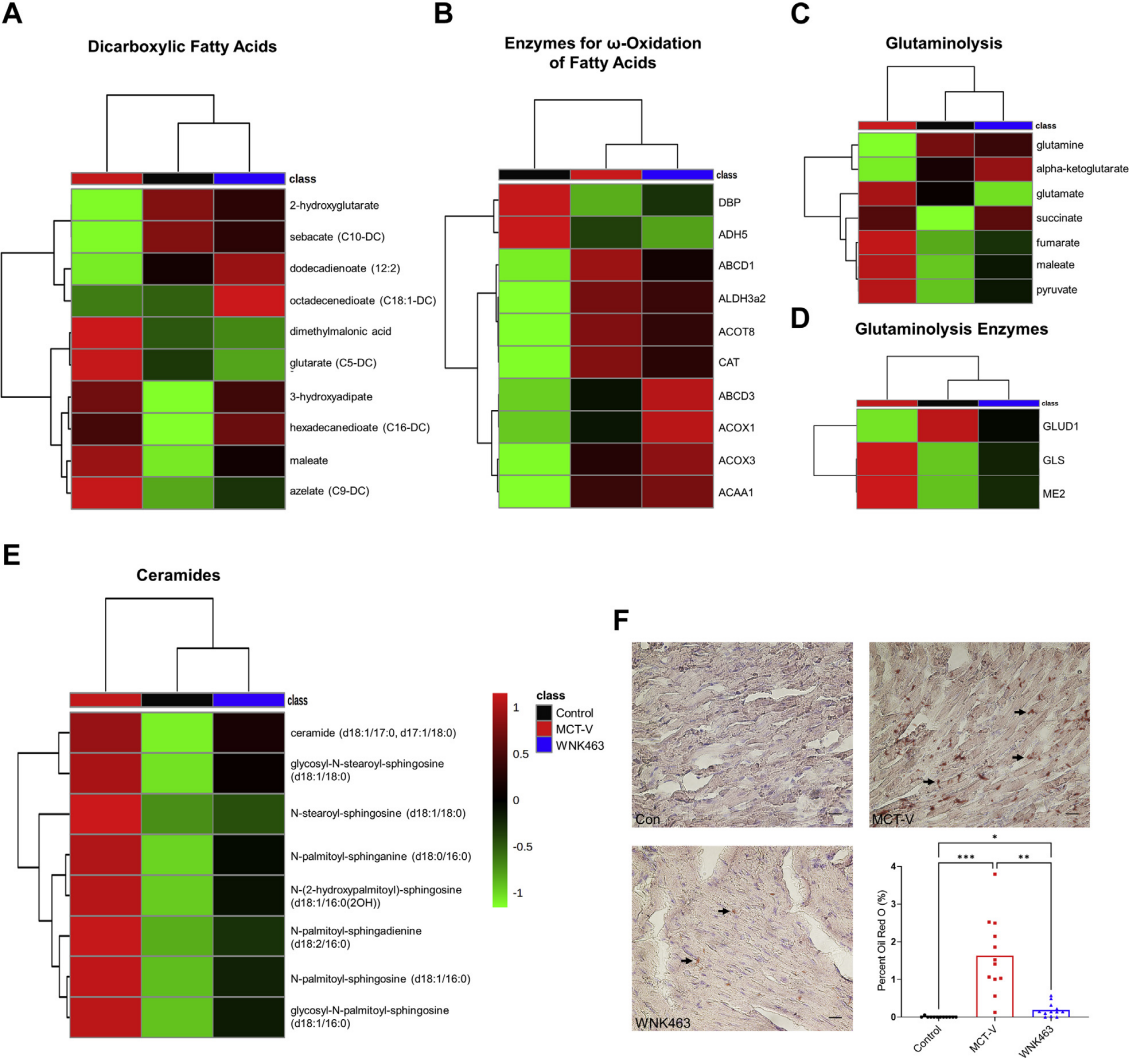
WNK463 Prevents Medium Chain DCA Accumulation, Reduces Signs of Glutaminolysis Induction, and Combats Lipotoxicity in the RV WNK463 prevents accumulation of **(A)** multiple dicarboxylic fatty acids (DCAs), **(B)** without modulating enzymes responsible for ω-oxidation of DCA. There are elevated levels of **(C)** glutaminolysis intermediates and **(D)** glutaminolysis enzymes in MCT-V RVs, which WNK463 normalizes. There is lipotoxicity in **(E)** MCT-V as demonstrated by higher levels of multiple ceramide species and **(F)** RV intramyocardial lipid deposition as assessed by Oil Red O staining. WNK463 counteracts these pathological changes (control rats: 0.005 ± 0.02%; MCT-V: 1.6 ± 1.0%; WNK463: 0.2 ± 0.2%; *P =* 0.001 between MCT-V and WNK463; n = 4 areas analyzed per RV and 3 RVs per group; 12 total RV areas analyzed per group). Scale bar 20 μm. ∗*P <* 0.05; ∗∗*P <* 0.01; ∗∗∗*P <* 0.001 by Brown-Forsythe and Welch ANOVA with Dunnett’s multiple comparisons test. Other abbreviations as in Figure 1.

To determine what metabolic pathway was most disrupted in RVD, we integrated our proteomics and metabolomics data using joint pathway analysis. The 3 most dysregulated pathways included fatty acid degradation, the TCA cycle, and pyruvate metabolism (Supplemental Figure 4). Because other groups have also demonstrated disrupted lipid metabolism in RVD because of PAH (28, 29, 30), we focused our efforts on understanding how 3 secondary metabolic consequences of altered mitochondrial FAO (ω-FAO, glutaminolysis, and lipotoxicity/ceramide accumulation) (Figure 5) were affected by WNK463. First, dicarboxylic fatty acid (DCA) levels were higher in MCT-V and MCT-WNK463 RVs than those in control RVs (Figure 6A), which suggested heightened ω-FAO in MCT-V and MCT-WNK463 RVs. However, MCT-WNK463 RVs had lower medium chain DCA levels compared with MCT-V RVs. Our proteomics experiments revealed upregulation of many proteins responsible for ω-FAO and peroxisomal degradation of DCAs in both MCT-V and MCT-WNK463 RVs (Figure 6B). These data supported the hypothesis that there was more ω-FAO in the setting of RV pressure overload, but the enhancement of mitochondrial FAO in MCT-WNK463 rats might have prevented accumulation of medium-chain DCAs. Second, there were elevated levels of multiple glutaminolysis metabolites (glutamate, succinate, fumarate, malate, and pyruvate) (Figure 6C) and the mitochondrial glutaminolysis enzymes, glutaminase, and malic enzyme 2 (Figure 6D) in the RVs of MCT-V rats, which indicated potential glutaminolysis induction. In contrast, WNK463 prevented the upregulation of glutaminase and malic enzyme 2 and accumulation of several glutaminolysis metabolites (Figures 6C and 6D). Third, metabolomics profiling revealed increased abundance of 8 species of ceramides, dihydroceramides, and hexosylceramides in the MCT-V RVs, but WNK463 depressed concentrations of all 8 lipids (Figure 6E). Finally, we observed ectopic lipid accumulation in MCT RVs, which WNK463 corrected (Figure 6F). In summary, these data suggest WNK463 combated accumulation of medium-chain DCAs, glutaminolysis induction, and lipotoxicity.

### WNK463 did not alter pulmonary vascular disease severity

**Figure 7:**
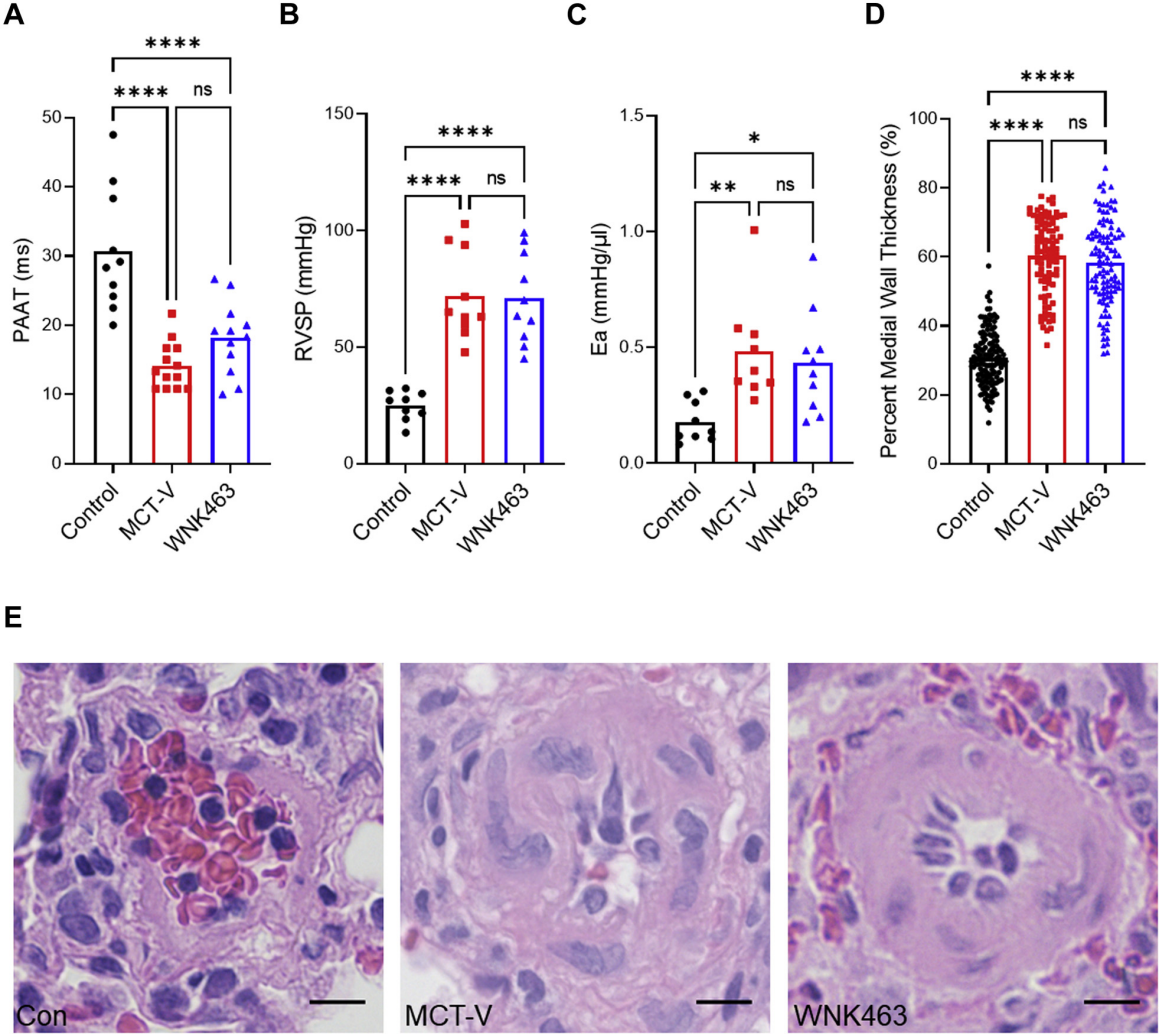
WNK463 Does Not Alter Pulmonary Vascular Disease Severity **(A)** There is no statistical difference in pulmonary artery acceleration time (PAAT) between MCT-V and WNK463 as assessed by echocardiography (control rats: 31 ± 9; MCT-V: 14 ± 3; WNK463: 18 ± 5 ms; *P =* 0.22 between MCT-V and WNK463; n = 10 control rats; n = 13 MCT-V; n = 12 WNK463). **(B)** No change in RV systolic pressure (RVSP) between MCT-V and WNK463 (control rats: 25 ± 6; MCT-V: 72 ± 19; WNK463: 71 ± 19 mm Hg; *P =* 0.99 between MCT-V and WNK463; n = 9 control rats; n = 10 MCT-V; n = 10 WNK463). **(C)** No difference in effective arterial elastance (Ea) between MCT-V and WNK463 (control rats: 0.18 ± 0.09; MCT-V: 0.48 ± 0.22; WNK463: 0.43 ± 0.22 mm Hg/μL; *P =* 0.84 between MCT-V and WNK463; n = 9 control rats; n = 9 MCT-V; n = 10 WNK463). **(D)** Histologically, there was no change in pulmonary small arteriole remodeling between MCT-V and WNK463 (control rats: 31 ± 8%; MCT-V: 60 ± 11%; WNK463: 58 ± 12% medial wall thickness; *P =* 0.52, n = 134 control rats; n = 94 MCT-V; n = 105 WNK463 pulmonary arterioles from 4 lung tissues per group). **(E)** Representative hematoxylin and eosin images of pulmonary arterioles. Scale bar 10 μm. ∗*P <* 0.05; ∗∗*P <* 0.01; ∗∗∗∗*P <* 0.0001; NS by 1-way ANOVA with Tukey’s multiple comparisons test in **A to C** or Brown-Forsythe and Welch ANOVA with Dunnett’s multiple comparisons test in **D**. Abbreviations as in Figure 1.

Next, we evaluated how WNK463 regulated pulmonary vascular disease severity to determine if our molecular changes were caused by modulation of RV afterload. Echocardiography showed WNK463 did not significantly prolong pulmonary artery acceleration time, an echocardiographic marker of pulmonary vascular disease severity (31), compared with MCT-V (Figure 7A). PV loop analysis demonstrated WNK463 did not change RV afterload because RV systolic pressure (Figure 7B) and effective arterial elastance (Figure 7C) were equivalent to MCT-V. Finally, pathological remodeling of the pulmonary arterioles was equivalent in MCT-V and MCT-WNK463 specimens (Figures 7D and 7E). Collectively, these data implied that the observed corrections in RV metabolism were not caused by less severe pulmonary vascular disease.

### WNK463 decreased RV hypertrophy and enhanced RV systolic and diastolic function

**Figure 8:**
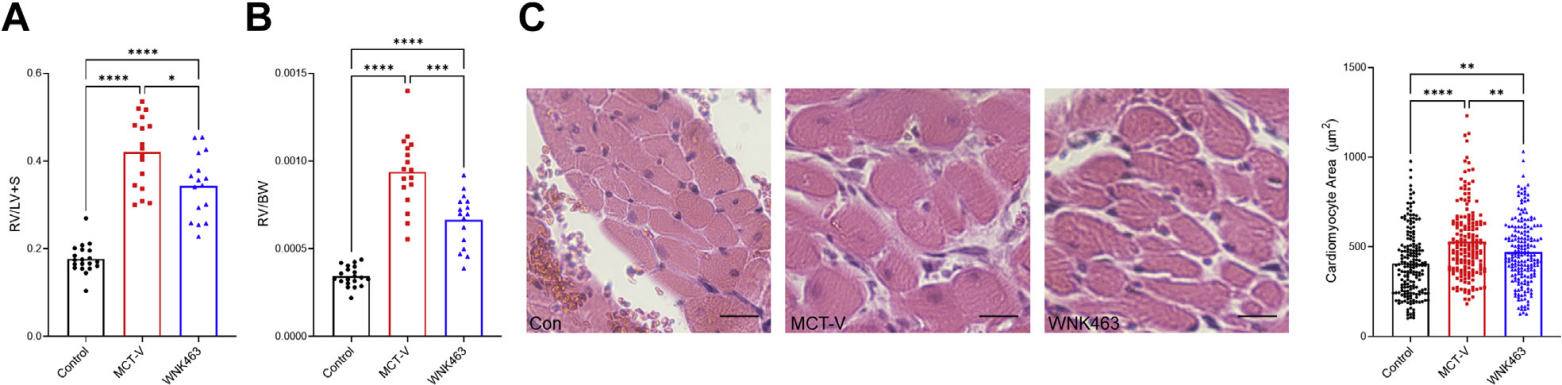
WNK463 Combats Pathological RV Hypertrophy Compared with MCT-V, WNK463 reduces **(A)** the Fulton index (control rats: 0.18 ± 0.03; MCT-V: 0.42 ± 0.08; WNK463: 0.34 ± 0.07; *P =* 0.02 between MCT-V and WNK463; n = 20 control rats; n = 17 MCT-V; n = 16 WNK463). **(B)** RV weight normalized to body weight (control rats: 0.0003 ± 0.00006; MCT-V: 0.0009 ± 0.0002; WNK463: 0.0007 ± 0.0002; *P =* 0.0004 between MCT-V and WNK463; n = 20 control rats; n = 17 MCT-V; n = 16 WNK463), and cardiomyocyte cross-sectional area (control rats: 408 ± 186; MCT-V: 530 ± 200; WNK463: 470 ± 180 μm^2^; *P =* 0.007 between MCT-V and WNK463; n = 184 control rats; n = 177 MCT-V; n = 196 WNK463 cardiomyocytes measured from 3 RVs per group) with representative images and quantification in **C**. Scale bar 20 μm. ∗*P <* 0.05; ∗∗*P <* 0.01; ∗∗∗*P <* 0.001; ∗∗∗∗*P <* 0.0001 by Brown-Forsythe and Welch ANOVA with Dunnett’s multiple comparisons test in **A and B** or 1-way ANOVA with Tukey’s multiple comparisons test in **C**. Abbreviations as in Figure 1.

WNK463 decreased the Fulton index (ratio of RV to LV and septum weight) (Figure 8A) and ratio of RV free wall weight to total body weight (Figure 8B). Furthermore, WNK463 reduced the RV cardiomyocyte cross-sectional area, but it was not completely normalized (Figure 8C). Thus, WNK463 mitigated pathological RV hypertrophy.

**Figure 9:**
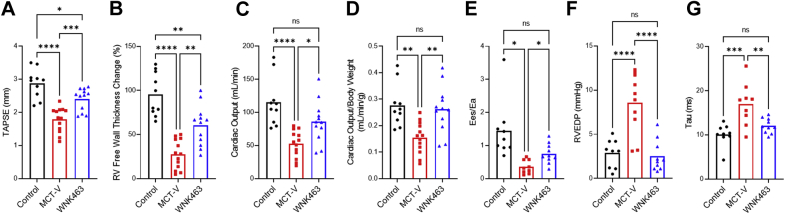
WNK463 Enhances RV Systolic and Diastolic Function WNK463 increases **(A)** tricuspid annular plane systolic excursion (TAPSE) (control rats: 2.9 ± 0.4; MCT-V: 1.8 ± 0.4; WNK463: 2.4 ± 0.3 mm; *P =* 0.0009 between MCT-V and WNK463; n = 10 control rats; n = 13 MCT-V; n = 12 WNK463), **(B)** percent RV free wall thickness change (control rats: 96 ± 24%; MCT-V: 28 ± 17%; WNK463: 60 ± 23%; *P =* 0.001 between MCT-V and WNK463; n = 10 control rats; n =13 MCT-V; n = 12 WNK463) **(C)** cardiac output (control rats: 115 ± 36; MCT-V: 52 ± 20; WNK463: 86 ± 31 mL/min; *P =* 0.02 between MCT-V and WNK463; n = 10 control rats; n = 13 MCT-V; n = 12 WNK463), and **(D)** cardiac output normalized to body weight (control rats: 0.28 ± 0.08; MCT-V: 0.15 ± 0.06; WNK463: 0.26 ± 0.09 mL/min/g; *P =* 0.004 between MCT-V and WNK463; n = 10 control rats; n = 13 MCT-V; n = 12 WNK463) as assessed by echocardiography. WNK463 augments RV-pulmonary artery coupling (Ees/Ea) (control rats: 1.4 ± 0.9; MCT-V: 0.4 ± 0.2; WNK463: 0.8 ± 0.3; *P =* 0.01 between MCT-V and WNK463; n = 9 control rats; n = 9 MCT-V; n = 10 WNK463), **(E)** reduces RV end-diastolic pressure (RVEDP) (control rats: 3 ± 2; MCT-V: 9 ± 3; WNK463: 3 ± 2 mm Hg; *P <* 0.0001 between MCT-V and WNK463; n = 9 control rats; n = 10 MCT-V; n = 10 WNK463) **(F)**, and improves RV diastolic function (tau) (control rats: 10 ± 2; MCT-V: 17 ± 5; WNK463: 12 ± 2 ms; *P =* 0.008 between MCT-V and WNK463; n = 9 control rats; n = 9 MCT-V; n = 10 WNK463) **(G)** when evaluated by closed-chest pressure-volume (PV) loop analysis. The volume data were not of high enough quality for one of the MCT-V rats, so Ees/Ea and RV tau were not obtained. No data were excluded from analysis. ∗*P <* 0.05; ∗∗*P <* 0.01; ∗∗∗*P <* 0.001; ∗∗∗∗*P <* 0.0001; NS by 1-way ANOVA with Tukey’s multiple comparisons test in **A to D** and **F and G** or Brown-Forsythe and Welch ANOVA with Dunnett’s multiple comparisons test in **E**. Other abbreviations as Figure 1.

We then used echocardiography and PV loops to examine RV function. Echocardiographic analysis demonstrated WNK463 increased tricuspid annular plane systolic excursion, percent RV free wall thickening, cardiac output, and cardiac output normalized to body mass (Figures 9A to 9D) compared with MCT-V. PV loop analysis showed end-systolic elastance/effective arterial elastance, the gold standard of RV function (32), was higher in MCT-WNK463 than MCT-V RVs (Figure 9E). In addition to the augmented RV systolic function, WNK463 treatment enhanced RV diastolic function as determined by a reduction in RV end-diastolic pressure and RV τ (relaxation time) (Figures 9F and 9G).

### Hypochloremia was associated with exacerbated RV dysfunction in PAH

**Figure 10:**
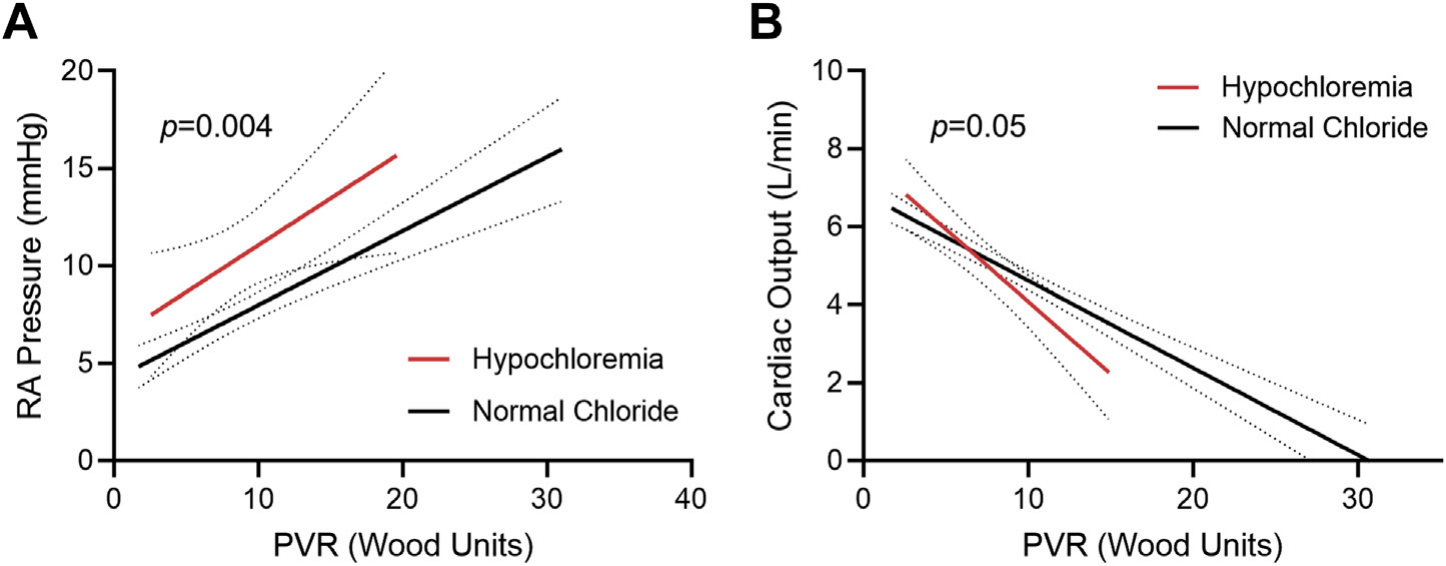
Hypochloremia Is Associated With Exaggerated RV Dysfunction in PAH **(A)** Patients with pulmonary arterial hypertension (PAH) with hypochloremia (serum chloride ≤101 mmol/L) have higher right atrial (RA) pressures at every pulmonary vascular resistance (PVR) compared with patients with PAH with normal serum chloride levels *(P =* 0.0004 between y-intercepts; *P =* 0.58 between slopes, n = 38 with hypochloremia and n = 162 with normal chloride). **(B)** Patients with PAH with hypochloremia have a more rapid descent in cardiac output as PVR increases *(P =* 0.05 between slopes; *P =* 0.56 between y-intercepts, n = 24 with hypochloremia and n = 131 with normal chloride). Abbreviation in Figure 1.

Finally, we analyzed the effects of hypochloremia, a condition that activates WNK1 (17), on RV function in a cohort of 217 patients with PAH (Supplemental Table 2). First, when we plotted the relationship between right atrial (RA) pressure and pulmonary vascular resistance (PVR), patients with hypochloremia had higher RA pressure at all PVR values than patients with normochloremia (Figure 10A). Furthermore, as PVR increased, patients with hypochloremia had a more rapid decline in cardiac output compared with patients with normal serum chloride levels (Figure 10B). These data associated hypochloremia with more severe RV dysfunction in patients with PAH.

## Discussion

In this study, we showed small molecule inhibition of WNK1 signaling prevented upregulation of GLUT1 and GLUT4 via mitigation of AS160 phosphorylation, which subsequently restored the levels of glucose metabolites in the RV. The normalization of RV glucose uptake by WNK463 depressed excess protein O-GlcNAcylation and glycation. In addition, WNK463 activated AMPK, normalized mitochondrial density, and restored and sometimes even increased levels of mitochondrial enzymes involved in the TCA cycle, the FAO pathway, and electron transport chain complexes. These proteomic changes were matched with metabolic shifts on metabolomics analysis that were indicative of partial correction of RV metabolism. Integration of our proteomics and metabolomics analyses identified FAO as the most altered pathway in preclinical RVD. WNK463 could combat the secondary consequences of impaired mitochondrial FAO in the dysfunctional RV because there were lower levels of glutaminolysis metabolites and enzymes, medium chain DCAs, and ceramides. These molecular changes resulted in enhanced RV systolic and diastolic function, which was not caused by to alteration in pulmonary vascular disease severity. Finally, hypochloremia in PAH resulted in more severe RVD, which provided evidence that this pathway might be relevant in human disease. In summary, these data identified WNK1 as a druggable target for PAH-associated RVD caused by modulation of glucotoxicity and subsequent metabolic derangements.

WNK463 prevented both protein O-GlcNAcylation and glycation, which resulted in less mitochondrial protein dysregulation and augmented metabolic function. Our results were congruent with several other studies that showed O-GlcNAcylation and glycation directly promoted mitochondrial dysfunction. There was evidence that these PTMs modulated mitochondrial function through multiple mechanisms. First, several mitochondrial proteins were O-GlcNAcylated (33, 34, 35) and glycated (36), and these PTMs could directly alter enzymatic activity. In addition, cardiac-specific transgenic overexpression of O-linked β-*N*-acetylglucosamine transferase, the enzyme that catalyzes the addition of O-GlcNAc, decreased mRNA levels of enzymes in FAO, the TCA cycle, and oxidative phosphorylation, which ultimately depressed mitochondrial function (37). Thus, excess O-GlcNAcylation might directly modulate enzymatic activity via PTM and depress transcription of enzymes in multiple mitochondrial metabolic pathways, which, in total, caused metabolic dysregulation. With regard to protein glycation, knockout of DJ-1, the protein that reverses protein glycation (38), reduced cardiomyocyte mitochondrial DNA content and resulted in cardiomyopathy (13), which suggested protein glycation could alter mitochondrial biogenesis and/or stability. Moreover, overexpression of DJ-1 mitigated excess protein glycation, which prevented the development of ischemia-reperfusion−induced LV failure (12). Thus, our results and other existing data showed O-GlcNAcylation and glycation adversely affected mitochondrial metabolic function and cardiac function.

Our data might provide a molecular explanation of the increased mortality associated with hypochloremia in PAH (14,15) and in patients with left heart failure (39, 40, 41). Based on our findings, we proposed hypochloremia activated WNK1, which subsequently heightened glucose uptake, which resulted in excess protein O-GlcNAcylation and glycation. These pathological PTMs caused mitochondrial metabolic dysfunction, which then increased the demand for glycolytic metabolism and cardiomyocyte glucose uptake, ultimately resulting in a vicious downward cycle that culminated in cardiac failure. However, WNK1-mediated glucotoxicity might be particularly important in RV failure because the Human Cardiac Cell Atlas revealed RV cardiomyocytes had higher expression levels of WNK1, GLUT4, and AS160 than LV cardiomyocytes (specifically, in cardiomyocyte population 2, which was more enriched in the RV than LV) (42) (Supplemental Table 2). In addition, RV cardiomyocytes exhibited higher rates of glycolysis than LV cardiomyocytes (43), which suggested alterations in glucose metabolism might have heightened consequences in the RV.

Our findings also provided important insights into the interplay of multiple metabolic pathways implicated in RV dysfunction in PAH. First, we showed WNK463 restored FAO in the RV, marked by increased levels of multiple acylcarnitine species (Figure 4C). RV acylcarnitines were reduced in human PAH (28), which suggested our results were directly relevant to human disease. Furthermore, we demonstrated ceramide accumulation in RV failure, a finding also observed in human PAH (28,29). Consistent with our data, elevated lipid deposition in the RV corresponded with RV hypertrophy and systolic and diastolic dysfunction in preclinical and human studies (29,44). Moreover, our data suggested ω-FAO was accentuated in rodent RVD (Figure 6B). Human studies also suggested heightened ω-FAO in PAH as octadecanedioate, a long chain DCA, was elevated in patients with PAH (45). Moreover, levels of octadecanedioate were inversely associated with RV function in pediatric PAH (46), providing further support that RVD was associated with excess ω-FAO. Finally, we provided evidence that glutaminolysis might be induced in RVD, which WNK463 prevented. Inhibition of glutaminolysis enhanced RV function in preclinical PAH, and there were higher levels of SLC1A5, the protein that imports glutamine into the cell, in human PAH RV specimens (47). In summary, we and others identified multiple metabolic derangements in the failing RV, and there appeared to be a highly interdependent relationship between defective mitochondrial FAO, induction of ω-FAO, and glutaminolysis and lipotoxicity. Disturbances in all of these metabolic pathways were observed in human studies, which suggested our results have direct human relevance.

Peroxisomes are secondary metabolic organelles that are responsible for metabolism of long chain and complex fatty acids (48), but the role of peroxisomes in cardiac dysfunction is understudied. The importance of peroxisomes in proper cardiac function is demonstrated by cardiomyopathy caused by cardiomyocyte-specific knockout of peroxisome proliferator-activated receptor-γ (49). Moreover, the peroxisome proliferator-activated receptor-γ agonist, pioglitazone enhanced FAO in isolated RV cardiomyocytes and rescued RVD in Sugen-hypoxia rats (6), albeit in the setting of reduced PAH severity. Our proteomics analysis revealed an increase in multiple peroxisomal proteins, and confocal microscopy showed peroxisome density and size were also elevated in RV failure (Supplemental Figure 5), which suggested compensatory peroxisome biogenesis occurred in RV pressure overload. Surprisingly, multiple peroxisomal proteins were lower in abundance in the RV than in the LV (48). This implied there might be chamber-specific differences in peroxisomal importance, and that the RV might have less of a peroxisomal reserve than the LV. However, future studies are needed to clearly delineate the role of peroxisomes in RVD.

Although the rationale to inhibit WNK1 signaling in our study was based on a clinical observation, other molecular mechanisms likely promote WNK1 upregulation in RVD. First, WNK1 levels were increased by tumor necrosis factor-α via reducing expression of neuronal precursor cell-expressed developmentally downregulated 4-2 E3-ubiquitin ligase, a protein that degrades WNK1 in kidney cells (50). This was directly relevant to RVD because tumor necrosis factor-α levels increase with the severity of RVD in rodent PAH (51). Moreover, aldosterone post-transcriptionally enhances WNK1 expression via miR-192 in the kidney (52). Again, this might be pertinent to RVD from PAH because serum aldosterone levels are inversely associated with cardiac output in human PAH (53). Thus, multiple pathways likely converge to promote WNK1 upregulation/activation and subsequent glucotoxicity in RVD, which might explain why WNK inhibition was so efficacious.

### Study limitations

Our study had important limitations that must be acknowledged. First, all of our animal studies were performed in male MCT rats because we wanted to use the most severe model of RV failure to probe WNK1 signaling. Moreover, we only used 1 model of PAH and RV failure, but as described previously, many of the metabolic disturbances we documented are also present in human analyses, so we believed this model was directly relevant to human RV failure. At this time, it is uncertain whether MCT models are hypochloremic, if hypochloremia is the sole cause of WNK1 upregulation, and whether there is increased activity of WNK1 in the RV of human patients with PAH with hypochloremia. The beneficial effects of WNK463 could be caused by inhibition of other isoforms of WNK, but we believed it was predominantly mediated by WNK1 because we were unable to detect WNK2 in cardiac extracts (Supplemental Figure 6). This was consistent with low WNK2 cardiac abundance observed in the Human Protein Atlas (54). In addition, WNK3 and WNK4 mRNA was not detected in cardiac tissue (17), which further supported the hypothesis that WNK1 was the most important WNK isoform for cardiac physiology. There might have been differences in mitochondrial robustness that led to variability in efficacy extraction in our proteomics analysis. However, that was less likely because most of the mitochondrial proteins were actually higher in the diseased animals, including multiple mitochondrial membrane and ribosomal proteins (Supplemental Figure 7). The reduction in RV end-diastolic pressure with WNK463 could be caused by the diuretic effects of the compound (25), but the normalization of RV τ suggested a true change in RV diastolic function. Finally, the increased abundance of hexosamine biosynthetic pathway intermediates might have affected RV physiology independent of protein O-GlcNAcylation because UDP-GlcNAc was used to synthesize extracellular matrix components (55). Consistent with this possibility, we detected a reduction in RV fibrosis in WNK463-treated rats (Supplemental Figure 8), which might be another mechanism underlying improvements in RV systolic and diastolic function with WNK463.

Perspectives **COMPETENCY IN MEDICAL KNOWLEDGE:** Low chloride is associated with increased mortality in PAH, but a mechanistic explanation is lacking. We showed inhibition of the chloride-activated protein; WNK1 restores RV function by modulating metabolism in rodent PAH.

**TRANSLATIONAL OUTLOOK:** Our data identified WNK1 as a potential druggable target for PAH-associated RVD. Further studies examining the safety and tolerability of WNK inhibition are needed to determine if our results could be translated to patients with PAH with RV failure.

## Conclusions

Our proteomics and metabolomics data showed small-molecule inhibition of WNK1 restructured RV mitochondrial protein regulation and metabolism in rodent PAH. WNK463 treatment augmented RV function without significantly altering PAH severity. Thus, WNK1 signaling may be a pharmacological target to enhance RV function, a currently untreatable and lethal consequence of PAH.

## Funding Support and Author Disclosures

Dr Prisco was supported by the National Institutes of Health (NIH) (F32 HL154533 and T32 HL144472), a University of Minnesota Clinical and Translational Science award (NIH UL1 TR002494), and a University of Minnesota Medical School Academic Investment Educational Program Grant. Dr Thenappan was supported by the Cardiovascular Medical Research and Education Fund and the University of Minnesota Futures Grant. Dr Prins was supported by National Institutes of Health (K08 HL140100), the Cardiovascular Medical Research and Education Fund, a Lillehei Heart Institute Cardiovascular Seed Grant, the University of Minnesota Faculty Research Development Grant, the United Therapeutics Jenesis Award, and an American Lung Association Innovative Award (IA-816386). Dr Thenappan has served on advisory boards for Actelion, United Therapeutics, Altavant Sciences, and Aria CV; and has received research funding for clinical trials from United Therapeutics, Aria CV, Gossimer Bio, and Acceleron. Dr Prins has served on advisory boards for Actelion and Edwards; and has received grant funding from United Therapeutics. All other authors have reported that they have no relationships relevant to the contents of this paper to disclose.
